# Genomic Studies in a Large Cohort of Hearing Impaired Italian Patients Revealed Several New Alleles, a Rare Case of Uniparental Disomy (UPD) and the Importance to Search for Copy Number Variations

**DOI:** 10.3389/fgene.2018.00681

**Published:** 2018-12-21

**Authors:** Anna Morgan, Stefania Lenarduzzi, Stefania Cappellani, Vanna Pecile, Marcello Morgutti, Eva Orzan, Sara Ghiselli, Umberto Ambrosetti, Marco Brumat, Poornima Gajendrarao, Martina La Bianca, Flavio Faletra, Enrico Grosso, Fabio Sirchia, Alberto Sensi, Claudio Graziano, Marco Seri, Paolo Gasparini, Giorgia Girotto

**Affiliations:** ^1^Department of Medicine, Surgery and Health Sciences, University of Trieste, Trieste, Italy; ^2^IRCCS Materno Infantile Burlo Garofolo, Trieste, Italy; ^3^Audiologia, Fondazione IRCCS Ca' Granda Ospedale Maggiore Policlinico, University of Milan, Milan, Italy; ^4^Medical Genetics Unit, Azienda Ospedaliero-Universitaria Città della Salute e della Scienza di Torino, Turin, Italy; ^5^Medical Genetics Unit, Department of Clinical Pathology, Azienda Unità Sanitaria Locale (AUSL) della Romagna, Cesena, Italy; ^6^Unit of Medical Genetics, S. Orsola-Malpighi Hospital, Bologna, Italy

**Keywords:** targeted re-sequencing, SNP arrays, hereditary hearing loss, italian families, molecular diagnosis

## Abstract

Hereditary hearing loss (HHL) is a common disorder characterized by a huge genetic heterogeneity. The definition of a correct molecular diagnosis is essential for proper genetic counseling, recurrence risk estimation, and therapeutic options. From 20 to 40% of patients carry mutations in *GJB2* gene, thus, in more than half of cases it is necessary to look for causative variants in the other genes so far identified (~100). In this light, the use of next-generation sequencing technologies has proved to be the best solution for mutational screening, even though it is not always conclusive. Here we describe a combined approach, based on targeted re-sequencing (TRS) of 96 HHL genes followed by high-density SNP arrays, aimed at the identification of the molecular causes of non-syndromic HHL (NSHL). This strategy has been applied to study 103 Italian unrelated cases, negative for mutations in *GJB2*, and led to the characterization of 31% of them (i.e., 37% of familial and 26.3% of sporadic cases). In particular, TRS revealed *TECTA* and *ACTG1* genes as major players in the Italian population. Furthermore, two *de novo* missense variants in *ACTG*1 have been identified and investigated through protein modeling and molecular dynamics simulations, confirming their likely pathogenic effect. Among the selected patients analyzed by SNP arrays (negative to TRS, or with a single variant in a recessive gene) a molecular diagnosis was reached in ~36% of cases, highlighting the importance to look for large insertions/deletions. Moreover, copy number variants analysis led to the identification of the first case of uniparental disomy involving *LOXHD1* gene. Overall, taking into account the contribution of *GJB2*, plus the results from TRS and SNP arrays, it was possible to reach a molecular diagnosis in ~51% of NSHL cases. These data proved the usefulness of a combined approach for the analysis of NSHL and for the definition of the epidemiological picture of HHL in the Italian population.

## Introduction

Hearing loss (HL) is a frequent disease affecting 1–3 in every 1,000 live births (Ječmenica et al., 2015). It is typically described based on its clinical presentation and can be classified by a number of different factors, such as the age of onset, severity, etiology, and pathobiology (Rehm and Morton, 1999; Dror and Avraham, 2009; Alford et al., 2014; Smith et al., 2014; Parker and Bitner-Glindzicz, 2015).

Overall, 60–70% of cases have a genetic etiology (Nishio et al., 2015) and can be further categorized as to whether the gene causes only hearing loss (non-syndromic or NSHL) or multiple clinical features (syndromic). NSHL accounts for the vast majority of hereditary HL cases and includes, according to the pattern of inheritance, autosomal recessive cases (~80%, labeled as “DFNB”), autosomal dominant (~20%, labeled as “DFNA”) X-linked or mitochondrial cases (<1%) (Stelma and Bhutta, 2014). In Italy, from 20 to 40% of cases are caused by mutations in *GJB2* gene (Cama et al., 2009; Primignani et al., 2009; Salvago et al., 2014), making it the major player. However, as expected, considering the unique and complex structure of the inner ear, many other genes have been found to be involved in the hearing phenotype. To date, 158 NSHL loci (60 DFNA loci, 88 DFNB loci, 6 X-linked loci, 2 modifier loci, 1 Y-linked locus, and 1 locus for auditory neuropathy), and 95 genes (27 DFNA genes, 56 DFNB genes, 8 DFNA/DFNB genes, and 4 X-linked genes) have been reported as causative (Hereditary Hearing Loss Homepage; http://hereditaryhearingloss.org/). In this light, a combined approach based on an accurate clinical characterization and on different analytical technologies represents the most effective strategy for the identification of the molecular cause of NSHL, which is essential for proper genetic counseling, recurrence risk estimation, prognosis, and therapeutic options.

Here, we report the results obtained on a large cohort of unrelated patients (*N* = 103), negative for *GJB2* mutations, applying a targeted re-sequencing panel of 96 HHL genes followed by SNP arrays in negative cases. This approach allowed: (1) the characterization of 31% of all NSHL cases, leading to an overall detection rate of ~51% (together with *GJB2*), (2) the identification of an extremely rare case of uniparental disomy (UPD) in HHL (i.e., the first one in *LOXHD1* gene), (3) the demonstration of the importance of copy number variants (CNVs), and (4) the identification of 17 new alleles.

## Materials and Methods

### Ethical Statement

All patients provided written informed consent forms for both genetic counseling and molecular genetic testing prior to enrolment. Written informed consent was obtained from the next of kin on behalf of the minors/children involved in this study. The study was approved by the Institutional Review Board of IRCCS Burlo Garofolo, Trieste, Italy. All research was conducted according to the ethical standard as defined by the Helsinki Declaration.

### Patients: Clinical Evaluation and Sample Collection

A total of 103 Italian unrelated NSHL patients have been recruited in the following centers (ENTs or Medical Genetics): Trieste (IRCCS Burlo Garofolo), Milano (IRCCS Cà Granda—Ospedale Maggiore Policlinico), Torino (A.O.U. Citta della Salute e della Scienza), Cesena (AUSL Romagna–Cesena), and Bologna (Policlinico S.Orsola-Malpighi). Inclusion criteria were: (1) absence of vestibular signs, (2) no obvious syndromic features, (3) absence of diabetes, cardiovascular diseases, visual problems, and neurological disorders, (4) absence of mutations in *GJB2, GJB6*, and *MTRNR1* genes.

All participants underwent pure tone audiometric testing (PTA) or auditory brainstem response (ABR) in order to characterize the severity of HL according to the International guidelines described by Clark (1981).

Based on the pedigree structure, cases were divided into sporadic (*N* = 57) and familial (*N* = 46), the latter being classified as autosomal recessive (AR) (*N* = 4), autosomal dominant (AD) (*N* = 14), X-linked dominant (*N* = 1), and Y-linked (*N* = 1). The remaining 27 cases had an unclear pattern of inheritance.

At least three to four individuals per family have been analyzed by sequencing (both affected and healthy), and for the sporadic cases both the proband and the parents have been sequenced.

### Targeted Resequencing (TRS) Hereditary Hearing Loss Panel

The 96 hearing loss genes panel described by Vozzi et al. has been used in this study (Vozzi et al., 2014). DNA libraries were constructed using Ion AmpliSeq^TM^ Library Kit 2.0 (Thermo Fisher Scientific), and run on the Ion Torrent Personal Genome Machine (PGM) System (Thermo Fisher Scientific), according to manufacturer's instructions.

### Data Analysis

TRS data were analyzed with Ion Torrent Suite^TM^ v4.0 software, set up with standardized parameters. Single Nucleotides Variations (SNVs) and Small Insertions and Deletions (INDELs) were collected into a standardized Variant Call Format (VCF) version 4.1 (Danecek et al., 2011). SNVs and INDELS were then annotated with ANNOVAR (Wang et al., 2010) using human genome build 19 (hg19) as the reference.

SNVs leading to synonymous amino acids substitutions not predicted as damaging and not affecting highly conserved residues were excluded, as well as SNVs/INDELs with quality score (QUAL) <20 and called in off-target regions.

A comparison between the identified genetic variants and data reported in NCBI dbSNP build150 (http://www.ncbi.nlm.nih.gov/SNP/) as well as in gnomAD (http://gnomad.broadinstitute.org/), and NHLBI Exome Sequencing Project (ESP) Exome Variant Server ^1^ led to the exclusion of those variants previously reported as polymorphism. In particular, a Minor Allele Frequency (MAF) cut off of 0.01 and one of 0.001 were used for recessive and dominant cases, respectively.

The pathogenicity of known genetic variants was evaluated using ClinVar (http://www.ncbi.nlm.nih.gov/clinvar/), Deafness Variation Database (http://deafnessvariationdatabase.org/) as well as The Human Gene Mutation Database (http://www.hgmd.cf.ac.uk/ac/index.php).

On the other hand, for novel variants, several *in silico* tools, such as PolyPhen-2 (Adzhubei et al., 2013), SIFT (Ng and Henikoff, 2003), MutationTaster (Schwarz et al., 2010), LRT (Chun and Fay, 2009), CADD score (Kircher et al., 2014) were used to evaluate the pathogenicity of the variant identified. Moreover, the evolutionary conservation of residues across species was evaluated by PhyloP (Pollard et al., 2010) and GERP (Cooper et al., 2005) scores.

Human Splicing Finder (HSF) version 2.4.1 (http://www.umd.be/HSF/) (Desmet et al., 2009) and Splice Site Prediction by Neural Network (NNSPLICE) version 9 (www.fruitfly.org) were used to predict the effect of the splice-site mutations.

We manually investigated the raw sequence reads for all the candidate pathogenic variants using the Integrative Genomics Viewer (IGV) (Thorvaldsdottir et al., 2013) with the purpose of excluding likely false positive calls due to read misalignment.

Finally, on a patient-by-patient basis, identified variants were discussed in the context of phenotypic data at interdisciplinary meetings and the most likely disease-causing SNVs/INDELs were analyzed by direct Sanger sequencing on a 3500 Dx Genetic Analyzer (Applied Biosystems), using ABI PRISM 3.1 Big Dye terminator chemistry (Applied Biosystems).

Sanger sequencing was employed also to perform the segregation analysis within the family.

### SNP Arrays Analysis

SNP arrays analysis was performed using the Human OmniExpress Exome-8 Bead Chip (Illumina Inc., San Diego, CA, United States) containing 960,919 loci derived from phases I, II and III of the International HapMap project. The array includes over 274,000 functional exonic markers, delivering unparalleled coverage of putative functional exonic variant selected from 12,000 individual exome and whole-genome sequences. A total of 200 ng of gDNA (50 ng/μl) for each sample was processed according to Illumina's Infinium HD Assay Super protocol. Normalization of raw image intensity data, genotype clustering, and individual sample genotype calls were performed using Illumina's Genome Studio software v2011.1 (cnvpartition 3.2.0). The CNVs were mapped to the human reference genome hg19 and annotated with UCSC RefGene. Allele detection and genotype calling were performed with Genome Studio software, B allele frequencies (BAFs) and log R ratios were exported as text files for PennCNV analysis (Wang et al., 2007).

### Protein Modeling and Molecular Dynamics Simulation

The protein structure for the modeling of two *de novo* variants of *ACTG1* was collected from the protein data bank (PDB, http://www.rcsb.org/pdb/) (PDB ID: 5JLH) (von der Ecken et al., 2016). The wild-type (WT) structure was used to generate the mutational models p.(Thr66Ile) and p.(Arg183Gln) with PyMOL (www.pymol.org). The WT and mutant structures were used in GROMACS (Groningen Machine for Chemical Simulation V 4.5.4 package) (Van Der Spoel et al., 2005; Hess et al., 2008) for molecular dynamics simulations. The GROMOS96 (van Gunsteren., 1996) force field was applied to the structures. Energy minimization was carried out using the steepest descent algorithm with a tolerance of 2,000 kJ/mol/nm. The energy-minimized structures were used for the molecular dynamics simulations. The SPC3 (Berendsen et al., 1981) water model was used for solvation in a cubic box (0.8 nm) with periodic boundary conditions applied in all directions. The simulation systems were neutralized by adding counter ions. A twin range cut-off was used for long-range interactions: 0.8 nm for van der Waals interactions and 1.4 nm for electrostatic interactions. All bond lengths were constrained with the LINCS algorithm (Hess et al., 1997). The SETTLE algorithm (Miyamoto and Kollman, 1992) was applied to constrain the geometry of the water molecules. The energy minimized system was subjected to 100 ps equilibration followed by 10 ns production molecular dynamics simulations with a time-step of 2 fs at constant temperature (300 K), pressure (1 atm) and number of particles, without any position restraints (Berendsen et al., 1984). The collected trajectories were analyzed using the tools within GROMACS and the structures were analyzed using PyMOL. In addition, the representative structures were selected from molecular dynamics simulations for the structural analyses using the cluster analysis (Krishnamoorthy et al., 2018) and surface mapping calculations.

## Results

### Targeted Re-sequencing (TRS)

For the 96 genes under investigation an average 95% of the targeted region was covered at least 20X, with a 337 mean-depth. A total of 170 Mbp of raw sequence data was produced per individual. We identified an average of 468 genetic variants (SNVs/INDELs) per subject. After applying the filtering pipeline described in the “Methods” section, an average of 17 residual SNVs/INDELs for each subject were obtained.

A likely pathogenic variant was found in 27 cases (15 familial out of 46 and 12 sporadic out of 57) (Table 1) leading to an overall detection rate of ~33% of the familial cases and ~21% of the sporadic ones. A summary of the phenotypic and genetic data of the TRS positive cases is reported in Tables 1,2 respectively.

**Table 1:** List of likely causative variants identified by TRS.

**Family ID**	**Locus**	**Gene**	**cDNA change**	**Amino acid change**	**Protein domain**	**gnomAD**	**GERP++**	**Polyphen-2**	**SIFT**	**Mutation Taster**	**CADD**	**References**
Familial case_1	DFNA2A	*KCNQ4* (NM_004700.3)	c.947G>T#(het)	p.(Gly316Val)	Transmembrane region	NA	5.27	D	D	D	26	NA
			c.1600A>G#(het)	p.(Ile534Val)	NA	NA	5.51	P	T	D	20.5	NA
Familial case_2	DFNA8/12	*TECTA* (NM_005422.2)	c.589G>A (het)	p.(Asp197Asn)	NIDO domain	NA	5.37	P	D	D	23	Hildebrand et al., 2011
Familial case_3	DFNA15	*POU4F3* (NM_002700.2)	c.690G>C (het)	p.(Arg230Ser)	POU domain	rs781045522; MAF: 4,063e-6	4.62	D	D	D	16.22	NA
Familial case_4	DFNA20/26	*ACTG1 (*NM_001199954.1)	c.847A>G (het)	p.(Met283Val)	ACTIN domain	NA	0.704	B	NA	D	13.71	NA
Familial case_5	DFNA22	*MYO6* (NM_004999.3)	c.599A>G (het)	p.(Asn200Ser)	MYSc ATPase domain	NA	4.98	D	D	D	23.4	NA
Familial case_6	DFNB16	*STRC* (NM_153700.2) *CATSPER2* (NM_172095.2)	deletion 49,23Kb 15q15.3		NA	NA	NA	NA	NA	NA	NA	Vona et al., 2015
Familial case_7	DFNA5	*DFNA5* (NM_001127454.1)	c.666_669delCTAC (het)	p.(Tyr223Serfs^*^49)	NA	NA	NA	NA	NA	D	NA	NA
Familial case_8	DFNA8/12	*TECTA* (NM_005422.2)	c.775G>C het)	p.(Gly259Arg)	NA	NA	4.92	D	D	D	24.6	Vozzi et al., 2014
Familial case_9	DFNB8/ 10	*TMPRSS3* (NM_024022.2)	c.1019C>G (het)	p.(Thr340Arg)	Tryp_SPc domain	NA	4.37	D	D	D	NA	Vozzi et al., 2014
			c.1291C>T (het)	p.(Pro431Ser)	Tryp_SPc domain	rs767931569, MAF: 2,437e-5	5.13	D	D	D	25.9	
Familial case_10	DFNB2	*MYO7A* (NM_000260.3)	c.1556G>A (het)	p.(Gly519Asp)	MYSc ATPase domain	rs111033206, MAF: 1,22e-5	5.02	D	D	D	19.77	Bharadwaj et al., 2000
			c.3670G>A (het)	p.(Ala1224Thr)	MyTH4 domain	rs748928605, MAF: 2,825e-5	5.31	D	D	D	31	NA
Familial case_11	DFNA11	*MYO7A* (NM_000260.3)	c.4268C>T (het)	p.(Thr1423Met)	B41 domain	rs779964645, MAF: 3,734e-5	4.85	P	T	D	19	NA
Familial case_12	DFNA4	*MYH14* (NM_001145809.1)	c.1150G>T (het)	p.(Gly384Cys)	MYSc domain	rs119103280, MAF: 0,002917	3.42	D	D	A	14.73	Donaudy et al., 2004
Familial case_13	DFNA8/12	*TECTA* (NM_005422.2)	c.6000-1G>T (het)	NA	NA	NA	NA	NA	NA	D	NA	NA
Familial case_14	DFNA6/14/38	*WFS1* (NM_006005.3)	c.2501G>A (het)	p.(Gly834Asp)	NA	NA	5.35	D	D	D	21.1	NA
Familial case_15	DFNX4	*SMPX* (NM_014332.2)	c.162delG (het)	p.(Lys55Serfs^*^25)	NA	NA	NA	NA	NA	NA	NA	NA
Familial case_16	DFNA22	*MYO6* (NM_004999.3)	deletion 75,8Kb 6q14		NA	NA	NA	NA	NA	NA	NA	
Familial case_17	DFNA48	*MYO1A* (NM_005379.3)	c.2468T>C ^denovo^ (het)	p.(Leu823Pro) ^denovo^	NA	NA	4.3	B	D	D	24.5	NA
Sporadic case_1	DFNB22	*OTOA* (NM_144672.3)	deletion 228,5Kb 16p12.2									Fontana et al., 2017
			c.1865T>A (hemizygous)	p.(Leu622His)	NA	rs750007142, MAF: 4,063e-6	4.77	D	D	D	19.03	
Sporadic case_2	DFNB8/10	*TMPRSS3* (NM_024022.2)	c.731G>A (het)	p.(Gly244Asp)	Tryp_SPc domain	rs397517377, MAF: 8,126e-6	5.33	D	NA	D	31	NA
			IVS4-6G>A (het)	NA	NA	rs374793617, MAF: 4,877e-5	NA	NA	NA	NA	NA	Scott et al., 2001
Sporadic case_3	DFNB4	*SLC26A4* (NM_000441.1)	c.1229C>T (het)	p.(Thr410Met)	NA	rs111033220, MAF: 0,0001844	5.1	D	D	D	23.6	Coyle et al., 1998
			c.2048T>C (het)	p.(Phe683Ser)	NA	MAF: 9,081e-6	5.42	D	D	D	20.8	Prasad et al., 2004
Sporadic case_4	DFNB77	*LOXHD1* (NM_144612.6)	c.3071A>G (UPD Chr 18)	p.(Tyr1024Cys)	LH2 domain	NA	4.53	D	D	D	14.99	NA
Sporadic case_5	DFNB3	*MYO15A* (NM_016239.3)	c.8090T>C (het)	p.(Val2697Ala)	NA	rs200451098, MAF: 0,0002671	5.19	D	D	D	22.2	Schrauwen et al., 2013
			c.8183G>A (het)	p.(Arg2728His)	NA	rs184435771, MAF: 0,0001915	5.34	D	D	D	26.9	Brownstein et al., 2011
Sporadic case_6	DFNA20/26	*ACTG1* (NM_001199954.1)	c.548G>A *^*denovo*^* (het)	p.(Arg183Gln) ^denovo^	ACTIN domain	rs781945750, MAF: 4,072e-6	3.57	P	D	D	17.61	NA
Sporadic case_7	NA	*PDZD7* (NM_001195263.1)	c.2850delC (het)	p.(Ser953Alafs^*^91)	NA	MAF: 0,0001122	NA	NA	NA	NA	NA	NA
			c.1841G>C (het)	p.(Arg614Thr)	NA	rs773503851, MAF: 2,092e-5	5.42	D	NA	D	27.1	NA
Sporadic case_8	DFNB21	*TECTA* (NM_005422.2)	c.6000-1G>A (het)	NA	NA	NA	NA	NA	NA	D	NA	NA
			c.2020C>T (het)	p.(Gln674^*^)	NA	NA	NA	NA	T	A	NA	NA
Sporadic case_9	DFNX2	*POU3F4* (NM_000307.4)	c.989G>A (hemizygous)	p.(Arg330Lys)	HOX domain	NA	5.07	D	D	D	25	NA
Sporadic case_10	NA	*PDZD7* (NM_024895.4)	c.329G>A (hom)	p.(Gly110Asp)	PDZ domain	NA	5	D	D	D	32	NA
Sporadic case_11	DFNB12	*CDH23* (NM_022124.5)	c.4562A>G (het)	p.(Asn1521Ser)	CA domain	rs780987516 MAF:1,804e-5	5.23	P	NA	D	25.2	Sloan-Heggen et al., 2015
			c.8377C>T (het)	p.(Arg2793Trp)	CA domain	rs749203752 MAF:4,068e-6	3.71	D	D	D	34	NA
Sporadic case_12	DFNB8/10	*TMPRSS3* (NM_024022.2)	c.718delC (het)	p.(His240Thrfs^*^35)	Tryp_SPc domain	NA	NA	NA	NA	NA	NA	NA
			c.579dupA (het)	p.(C194Metfs^*^17)	SR domain	NA	NA	NA	NA	NA	NA	Battelino et al., 2016; Lechowicz et al., 2017
Sporadic case_13	DFNA20/26	*ACTG1* (NM_001199954.1)	c.197C>T ^denovo^(het)	p.(Thr66Ile) ^denovo^	ACTIN domain	NA	3.99	B	NA	D	22	NA
Sporadic case_14	DFNX2	*POU3F4* (NM_000307.4)	c.967C>G (hemizygous)	p.(Arg323Gly)	HOX domain	rs104894924	4.14	D	D	D	16.53	Cremers et al., 2000
Sporadic case_15	DFNB16	*STRC* (NM_153700.2)	deletion 49kb 15q15.3		NA	NA	NA	NA	NA	NA	NA	

*All mutations have been classified based on their frequency reported in Genome Aggregation Database (gnomAD, http://gnomad.broadinstitute.org/) and their pathogenicity. In particular, the following tools have been used: GERP++ (higher number is more conserved), Polyphen-2 (D, Probably damaging; P, possibly damaging; B, benign), SIFT (D, deleterious; T, tolerated), MutationTaster “A” (“disease_causing_automatic”); “D” (“disease_causing”); “N”(“polymorphism”); “P” (“polymorphism_automatic”), and CADD (Pathogenicity score: > 10 predicted to be deleterious). NA, not available. DN, de novo mutation; #, mutation in cis*.

**Table 2:** Inheritance pattern and phenotypic data of all families and sporadic cases positive to TRS and/or SNP array.

**ID**	**Inheritance pattern**	**Type of HL**
Familial case_1	AR	Early-onset bilateral symmetric moderate NSHL
Familial case_2	AD	Early-onset bilateral symmetric mild to moderate NSHL
Familial case_3	AD	Early-onset bilateral symmetric moderate to severe NSHL
Familial case_4	AD	Adult-onset bilateral symmetric severe to profound NSHL
Familial case_5	AD	Early-onset bilateral symmetric severe to profound progressive NSHL
Familial case_6	AR	Early-onset bilateral symmetric moderate NSHL
Familial case_7	AD	Adult-onset bilateral symmetric moderate high frequencies NSHL
Familial case_8	AD	Early-onset bilateral symmetric moderate to severe NSHL
Familial case_9	AR	Early-onset bilateral symmetric moderate to profound medium-high frequencies NSHL (Sky-slope)
Familial case_10	AR	Early-onset bilateral symmetric moderate to severe NSHL
Familial case_11	AD	Early-onset bilateral symmetric moderate to severe NSHL
Familial case_12	AD	Early-onset bilateral symmetric mild to moderate progressive NSHL
Familial case_13	AD	Early-onset bilateral symmetric severe to profound NSHL
Familial case_14	AD	Early/adult-onset, bilateral symmetric moderate NSHL at the low and high frequencies
Familial case_15	XL	Early-onset bilateral symmetric severe to profound medium-high frequencies NSHL
Familial case_16	AD	Adult-onset bilateral symmetric moderate NSHL
Familial case_17	AD	Early-onset bilateral symmetric moderate to severe NSHL
Sporadic case_1	–	Early-onset bilateral symmetric severe to profound medium-high frequencies NSHL
Sporadic case_2	–	Early-onset bilateral symmetric severe to profound medium-high frequencies NSHL (Sky-slope)
Sporadic case_3	–	Early-onset bilateral symmetric severe to profound NSHL
Sporadic case_4	–	Early-onset bilateral symmetric severe to profound NSHL
Sporadic case_5	–	Early-onset bilateral symmetric profound NSHL
Sporadic case_6	–	Early-onset bilateral symmetric moderate to severe NSHL (Sky-slope)
Sporadic case_7	–	Early-onset bilateral symmetric moderate to severe NSHL
Sporadic case_8	–	Early-onset bilateral symmetric severe NSHL
Sporadic case_9	–	Early-onset bilateral symmetric moderate to severe NSHL
Sporadic case_10	–	Early-onset bilateral symmetric mild to moderate NSHL
Sporadic case_11	–	Early-onset bilateral symmetric profound NSHL
Sporadic case_12	–	Early-onset bilateral symmetric profound NSHL
Sporadic case_13	–	Early-onset bilateral symmetric mild to moderate NSHL
Sporadic case_14	–	Early-onset bilateral symmetric moderate to severe NSHL
Sporadic case_15	–	Early-onset bilateral symmetric moderate NSHL

*AR, autosomal recessive inheritance; AD, autosomal dominant inheritance; XL, X-linked pattern of inheritance; –, unclear pattern of inheritance. Hearing loss has been classified according to the following criteria: slight, 16–25 dB (dB); mild, 26–40 dB; moderate, 41–55 dB; moderately severe, 56–70 dB; severe, 71–90 dB; profound, 91 dB or more*.

**Figure 1:**
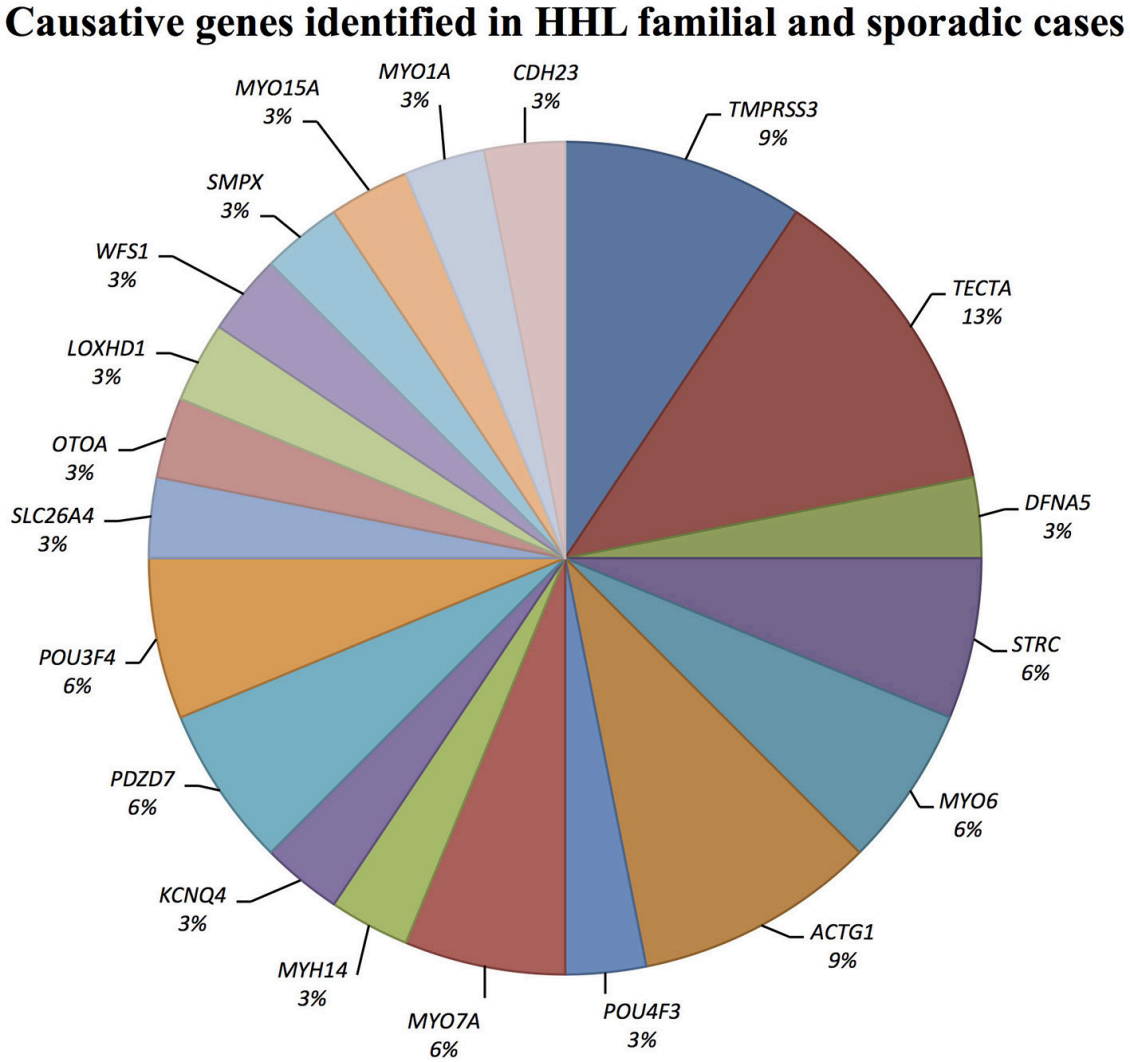
Causative genes identified in our cohort of Italian patients. The graph shows the distribution of the causative genes detected by TRS and SNP arrays.

Causative mutations in the following genes were identified: *ACTG1, CDH23, DFNA5, KCNQ4, LOXHD1, MYH14, MYO1A, MYO6, MYO7A, MYO15A, OTOA, PDZD7, POU3F4, POU4F3, SLC26A4, SMPX, TECTA, TMPRSS3*, and *WFS1* (Figure 1).

A detailed summary of pathogenic variants identified in our screening is reported in Table 1. Overall, 23 already known mutations and 16 novel variants have been detected as here detailed: 30 missense (two *de novo* in *ACTG1*, and one in *MYO1A*), four frameshift deletions, one frameshift insertion, one non-sense, three variants affecting splice-sites.

Moreover, since 14 familiar and sporadic cases were carrying only one causative allele (i.e., an already known pathogenic mutation or a variant predicted as highly damaging) in an autosomal recessive gene (Table 3), we hypothesized that a second mutation, or a large CNV, has been missed in trans. In this light, these individuals have been further investigated with SNP arrays and the most interesting results are reported below.

**Table 3:** List of likely damaging alleles identified by TRS in recessive genes.

**Family ID**	**Locus**	**Gene**	**cDNA change**	**Amino acid change**	**Protein domain**	**gnomAD**	**GERP++**	**Polyphen-2**	**SIFT**	**Mutation Taster**	**CADD**	**References**
Sporadic case_16	DFNB3	*MYO15A* (NM_0167239.3)	c.7305_7315dupGAGACCCCCAG (het)	p.(Glu2439Glyfs^*^35)	NA	NA	NA	NA	NA	D	NA	NA
Familial case_18	DFNB3	*MYO15A* (NM_0167239.3)	c.1634C>T (het)	p.(Ala545Val)	NA	rs199740747, MAF: 0,0009289	4.95	P	D	P	26.4	Sloan-Heggen et al., 2015
Sporadic case_17	DFNB1B	*GJB6* (NM_001110219.2)	c.209C>T (het)	p.(Pro70Leu)	CNX domain	rs727505123, MAF: 1.444e-5	5.38	D	D	D	21.8	NA
Familial case_19	DFNB77	*LOXHD1* (NM_144612.6)	c.4480C>T (het)	p.(Arg1494^*^)	NA	rs201587138, MAF: 0,0006741	NA	NA	T	A	NA	Diaz-Horta et al., 2012
Sporadic case_18	DFNB3	*MYO15A* (NM_016239.3)	c.3836A>G (het)	p.(Tyr1279Cys)	MYSc domain	NA	4.95	D	D	D	18.09	NA
Familial case_20	DFNB9	*OTOF* (NM_194248.2)	c.2374C>T (het)	p.(Arg792Trp)	Coiled coil region	rs148532589, MAF: 0,0009638	0.063	D	D	D	21.9	Sloan-Heggen et al., 2016
Sporadic case_19	DFNB12	*CDH23* (NM_022124.5)	c.512T>C (het)	p.(Phe171Ser)	CA Cadherin repeats	NA	5.43	D	D	D	28.9	NA
Sporadic case_20	DFNB24	*RDX* (NM_002906.3)	c.775C>T (het)	p.(Pro259Ser)	FERM_C domain	rs770505660, MAF: 4,079e-6	5.02	P	D	D	20.8	NA
Sporadic case_21	DFNB57	*PDZD7* (NM_001195263.1)	c.307G>C (het)	p.(Gly103Arg)	PDZ domain	rs148695069 MAF:1.624e-5	5.07	D	D	D	25.2	Booth et al., 2015
Familial case_21	DFNB36	*ESPN* (NM_031475.2)	c.935C>T (het)	p.(Ser312Leu)	NA	rs189442618 MAF:1,087e-4	4.08	P	D	D	26.6	NA
Sporadic case_22	DFNB24	*RDX* (NM_002906.3)	c.740A>C (het)	p.(Asn247Thr)	FERM_C domain	NA	5.02	B	D	D	27.6	NA
Sporadic case_23	DFNB35	*ESRRB* (NM_004452.3)	c.1205C>T (het)	p.(Thr402Met)	HOLI domain	NA	5.57	D	T	D	23.4	NA
Sporadic case_24	DFNB61	*SLC26A5* (NM_206883.2)	c.1322C>T (het)	p.(Ser441Leu)	Transmembrane region	rs773544931 MAF:1.624e-05	4.7	P	D	D	21.9	NA
Sporadic case_25	DFNB57	*PDZD7* (NM_001195263.1)	c.145C>T (het)	p.(Arg49Trp)	NA	NA	4.43	D	T	D	20.2	NA

*TRS led to the identification of four known causative, and ten potentially causative, heterozygous alleles in recessive genes. For all variants the affected gene, cDNA and protein change are indicated. All mutations have been classified based on their frequency reported in Genome Aggregation Database (gnomAD, http://gnomad.broadinstitute.org/) and their pathogenicity. In particular, the following tools have been used: GERP++ (higher number is more conserved), Polyphen-2 (D, Probably damaging; P, possibly damaging; B, benign), SIFT (D, deleterious; T, tolerated), MutationTaster “A” (“disease_causing_automatic”); “D” (“disease_causing”); “N” (“polymorphism”); “P” (“polymorphism_automatic”), and CADD (Pathogenicity score: > 10 predicted to be deleterious). NA, not available*.

#### TECTA

**Figure 2:**
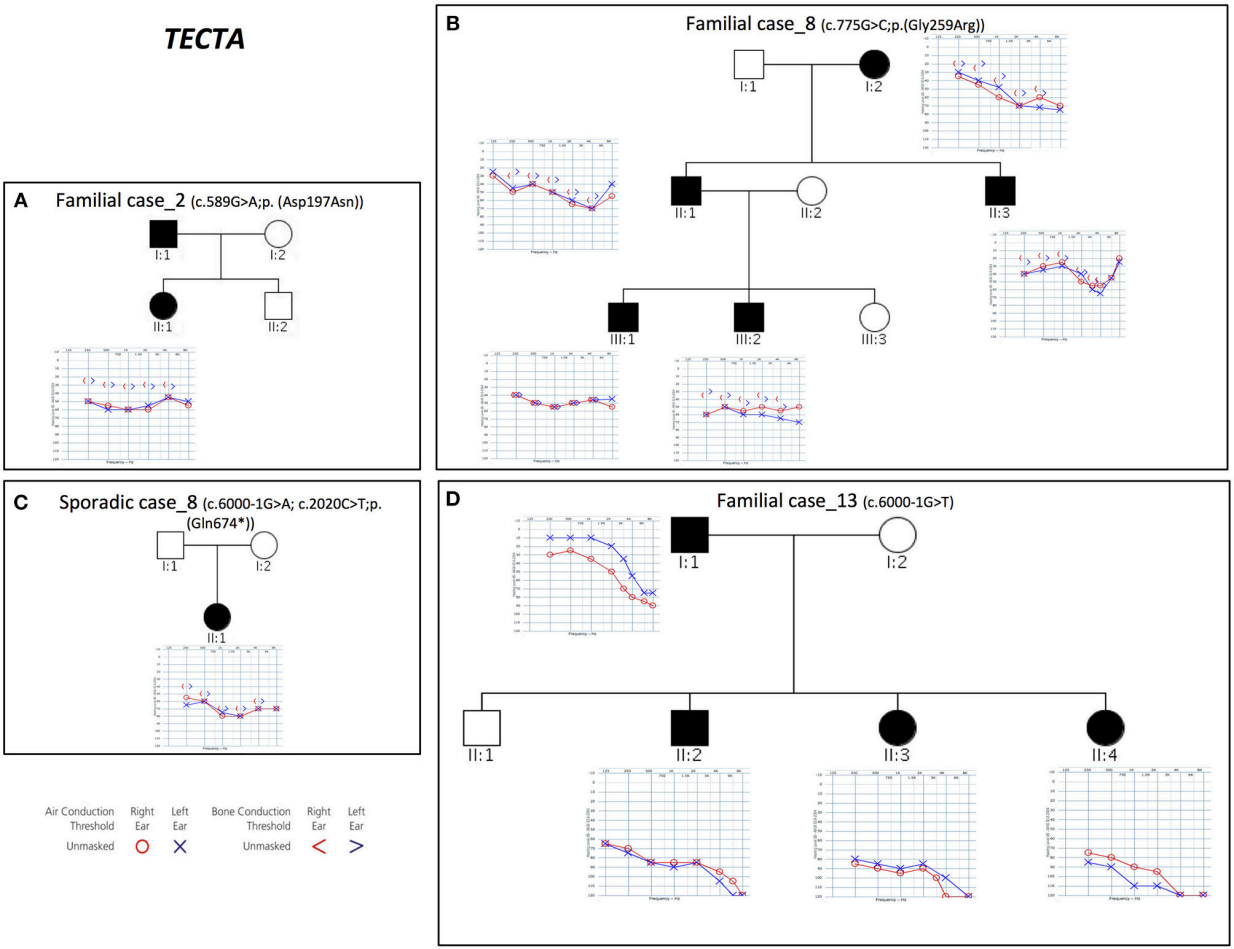
Pedigrees and audiograms of patients carrying variants in *TECTA* gene. **(A–D)** Phenotypic details of all patients carrying pathogenic variants in *TECTA* gene. Filled symbols represent affected individuals.

Combining previous data (Vozzi et al., 2014) with the results of the present study, *TECTA* (NM_005422.2) has been identified as the major NSHL gene in the Italian population (after *GJB2*), characterizing 13% of positive cases (with both autosomal dominant and recessive pattern of inheritance; Figure 2). Four different alleles in Familial case_8 [c.775G>C; p.(Gly259Arg)], Familial case_13 (c.6000-1G>T) and Sporadic case_8 [c.6000-1G>A; c.2020C>T; p.(Gln674^*^)] (Table 1), plus one already described [c.589G>A; p.(Asp197Asn)] in Vozzi et al. (Familial case_2) have been identified.

All patients show an early onset symmetrical NSHL, with different degrees of severity (Table 2 and Figure 2). The identified variants, predicted as damaging by several *in silico* predictor tools, affect functional domains of the α-tectorin (the entactin (ENT)-like, zonadhesin (ZA), and zona pellucida (ZP) domains) and involve highly conserved residues. Two of these changes [c.589G>A, p.(Asp197Asn); c.775G>C, p.(Gly259Arg)] were already known for causing NSHL, while the remaining three [c.6000-1G>T; c.6000-1G>A; c.2020C>T, p.(Gln674^*^)] have been identified in the present study.

Two different splicing variants affecting the same nucleotide have been identified in Familial case_13 and Sporadic case_8. Since *TECTA* gene is expressed at low levels in peripheral blood, it was not possible to test their effect on mRNA processing directly on the patients samples, however, according to the prediction of Human Splicing Finder software (http://www.umd.be/HSF3/) the c.6000-1G>T allele causes the total loss of the acceptor splice site, while the c.6000-1G>A leads to the loss of the acceptor splice site, together with the creation of a new site one nucleotide after.

#### ACTG1

**Figure 3:**
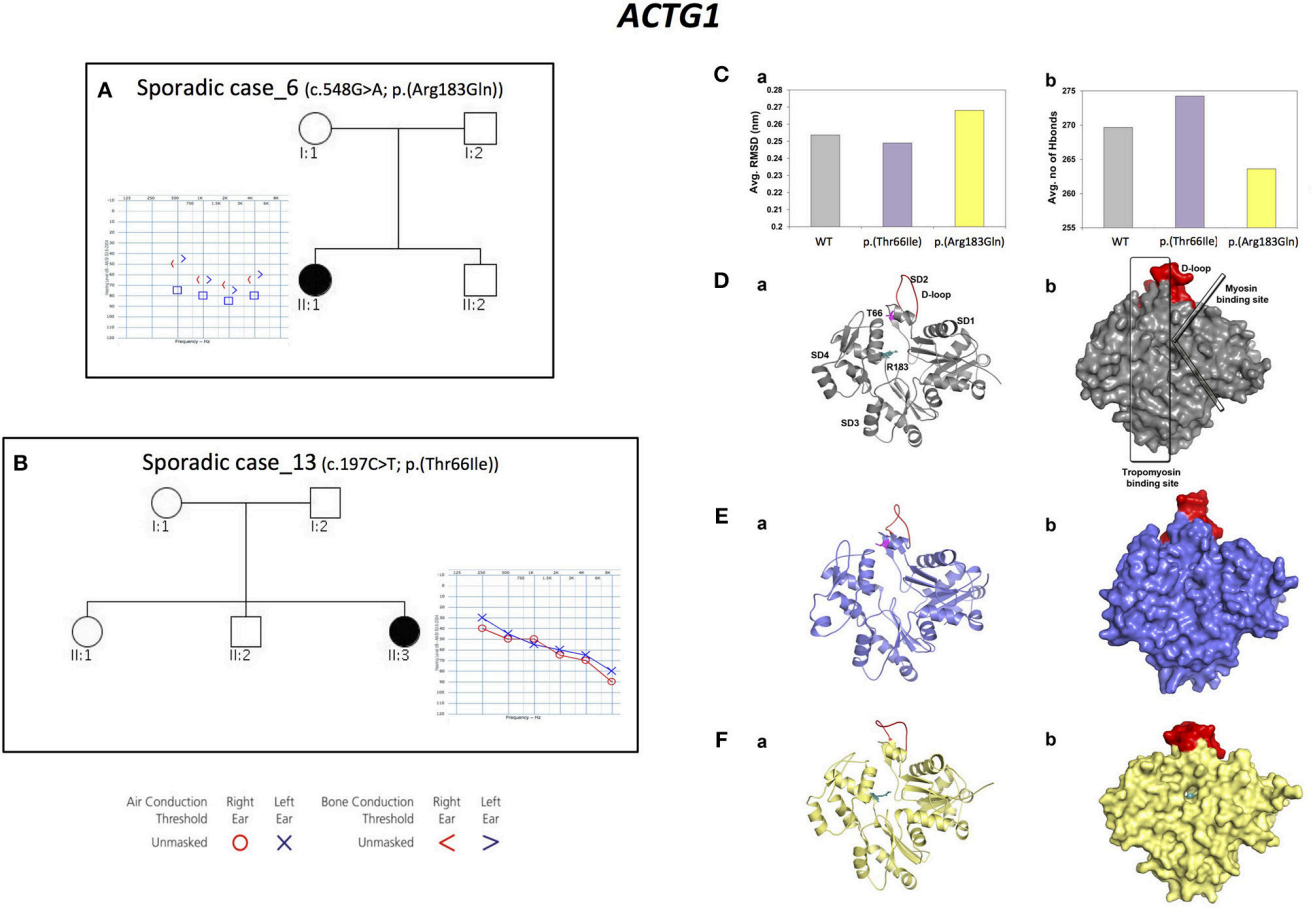
Phenotypic features of patients carrying variants in *ACTG1* gene and molecular modeling of *ACTG1 de novo* variants. **(A,B)** Pedigrees and audiograms of patients carrying *de novo* variants in *ACTG1*. Filled symbols represent affected individuals. **(C)** Trajectory based analyses of the WT and mutants (a) structural stability and (b) intra-molecular interactions, **(D–F)** (a) Cartoon and (b) surface models for the representative structures from molecular dynamics simulations. Here, WT is labeled with structural features and the p.(Thr66Ile) and p.(Arg183Gln) mutations are labeled as sticks colored in pink and teal, respectively. The structurally and functionally important D-loop is shown in red.

Three alleles in *ACTG1* (NM_001199954.1) have been identified: two are novel, [c.847A>G; p.(Met283Val) in Familial case_4, c.197C>T, p.(Thr66Ile) in Sporadic case_13] while one was already described [c.548G>A; p.(Arg183Gln) in Sporadic case_6]. All variants affect the actin-binding domain of ACTG1 protein. Patients display various degrees of severity of hearing loss (from mild-moderate, to severe-profound) and different age of onset (both adult-onset and early-onset), as expected from autosomal dominant NSHL (Table 2, Figure 3). Overall mutations in *ACTG1* characterize 11% of all cases in our cohort (Table 1).

Since *ACTG1 de novo* mutations are a rare cause of NSHL (Wang et al., 2018), protein modeling and molecular dynamics simulations of these two variants have been performed.

The structural stability of the WT and of the *ACTG1* mutants was dynamically calculated using root mean square deviation (RMSD). Results showed that the deviation pattern was different for the mutants compared to the WT (Figure 3C-a); in particular, the p.(Thr66Ile) mutant deviated less than the WT, while the p.(Arg183Gln) mutant deviated more than the WT. This behavior correlates with the average number of hydrogen bonds, which are needed for protein's structural stability (Figure 3C-b). Here, p.(Thr66Ile) displayed an increased number of hydrogen bonds compared to both the WT and the p.(Arg183Gln) mutant and this explains the stable/rigid structural nature of p.(Thr66Ile). On the other hand, the p.(Arg183Gln) mutant showed a decrease in the number of hydrogen bonds that might result in an increased protein flexibility (Figure 3C-a).

The representative structures from molecular dynamics simulations showed that the mutational spots for both Thr66 and Arg183 are located in the binding region of the tropomyosin (Figure 3D). Results showed that the mutant p.(Thr66Ile), which is very close to the D-loop, causes a conformational change of the loop itself that alters the binding surface, while in the case of p.(Arg183Gln), both the tropomyosin-binding region and the D-loop are involved leading to a potential cavity on the binding surface (Figures 3E,F).

These results proved that both variants affect a key-binding region, introducing few critical changes in the structure.

#### *POU4F3* and *SMPX*

**Figure 4:**
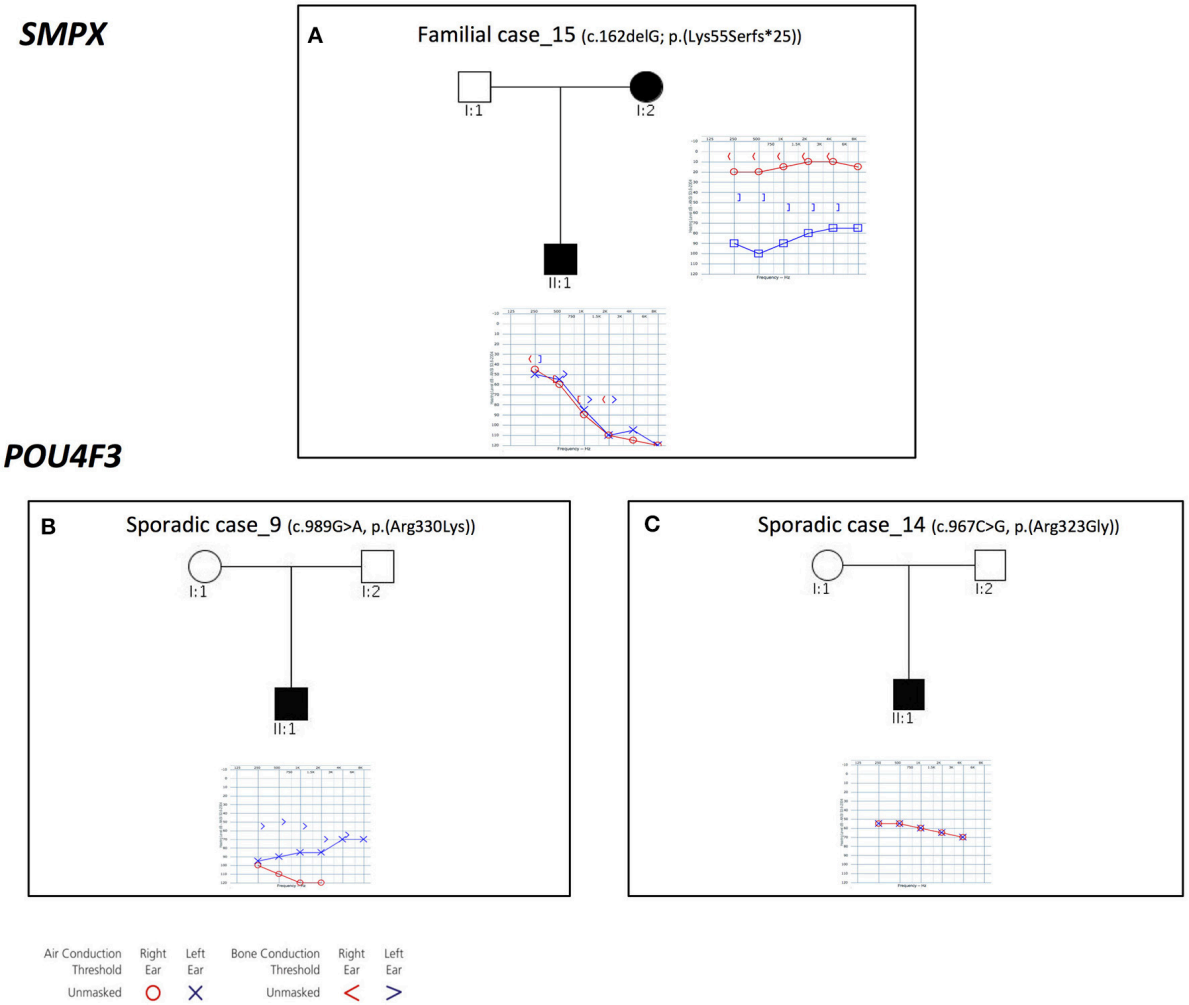
Pedigrees and audiograms of patients with X-linked HHL. **(A)** Patients of Familial case_15 carry a frameshift deletion in SMPX gene. **(B-C)** Sporadic case_9 and 14 carry two missense variant in *POU4F3* gene. Filled symbols represent affected individuals.

Three families with an X-linked pattern of inheritance have been detected (Figure 4).

Familial case_15 carries a novel frameshift deletion [c.162delG; p.(Lys55Serfs^*^25)] in *SMPX* gene (NM_014332.2), known for causing dominant X-linked NSHL (Table 1), The proband presents post-lingual bilateral symmetric severe to profound medium-high frequencies HL, while the mother shows a monolateral profound to severe HL (Figure 4A). As expected, no preferential X-inactivation has been detected in subject I:2.

*POU4F3* (NM_000307.4), known for causing recessive X-linked HL, was found to be mutated in Sporadic Case_9 (c.989G>A, p.(Arg330Lys) and Sporadic Case_14 (c.967C>G, p.(Arg323Gly) (Table 1). Both variants affect the HOX domain of the protein and are predicted to be damaging. In particular, the c.967C>G, p.(Arg323Gly) allele, was already known to cause hearing loss (Cremers et al., 2000). Both patients display early-onset bilateral symmetric moderate to severe hearing loss, with perilymphatic gusher occurred during cochleostomy (Figures 4B,C).

### SNP Arrays

Cases negative to TRS, or those showing a single variant in recessive genes, were analyzed with SNP arrays.

#### LOXHD1

**Figure 5:**
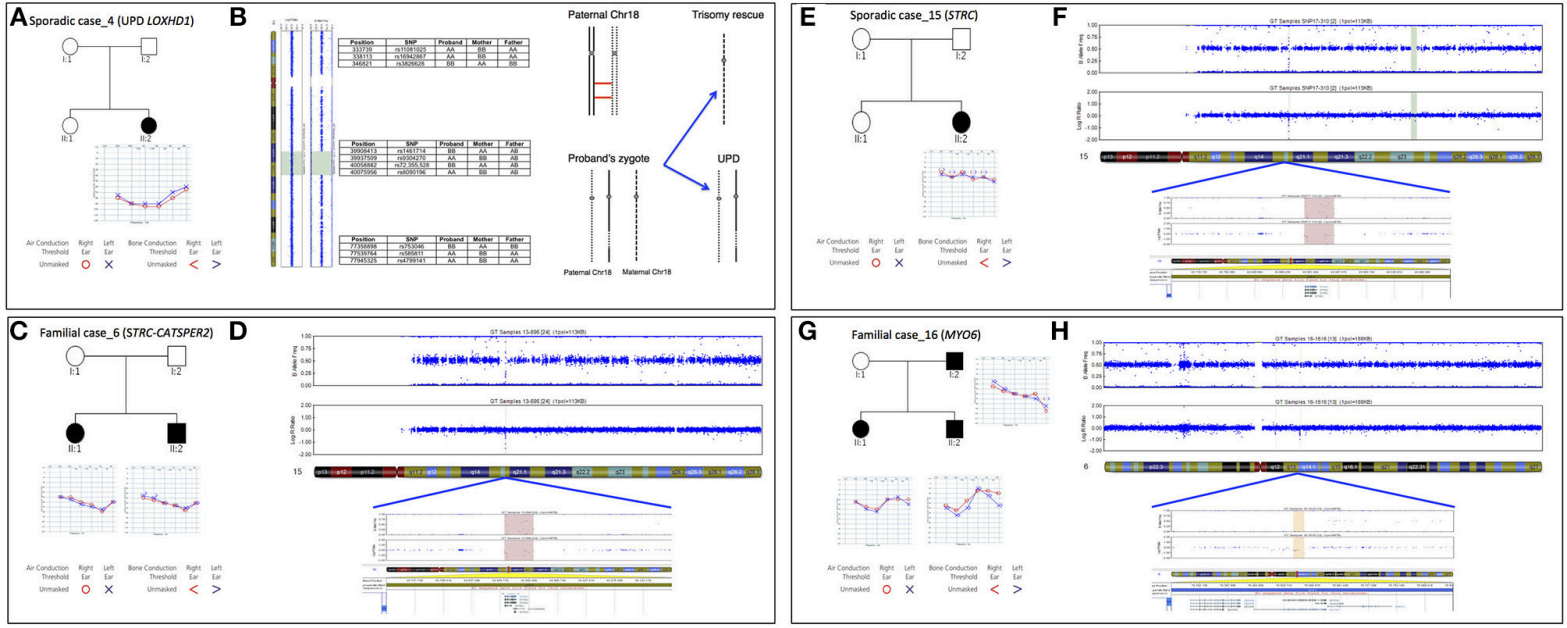
Pedigree, audiograms, and SNP array data of cases carrying pathogenic CNVs. **(A)** Pedigree and audiometric features of Sporadic case_4. Filled symbols represent affected individuals. **(B)** SNP array analysis of *LOXHD1* gene: results of SNP array on the proband show a UPD with ROH region (reported in green) suggesting a recombination event originated in meiosis I. The log R ratio of the analysis is consistent with normal copy number. Some of the markers in both the telomeric regions 18p and 18q plus in the ROH region are also indicated. **(C)** Pedigree and audiometric features of Familial case_6. Filled symbols represent affected individuals. **(D)** SNP array analysis: the image shows a double deletion (CNV = 0) of 49.23 Kb in the q15.3 region of chromosome 15 (highlighted in red). The deletion includes *STRC* and *CATSPER2* genes. **(E)** Pedigree and audiometric features of Sporadic case_15. Filled symbols represent affected individuals. **(F)** SNP array analysis: the graph shows a double deletion (CNV = 0) of 49 Kb in the q15.3 region of chromosome 15 (highlighted in red). Deletion includes *STRC* gene. **(G)** Pedigree and audiometric features of Familial case_16. Filled symbols represent affected individuals. **(H)** SNP arrays analysis: the image shows a deletion (CNV = 1) of 75.8 Kb in the q14.1 region of chromosome 6 (highlighted in orange). In the deletion region is possible to see a loss of heterozygosity in the B allele frequency and a lowering of the log ratio under the zero (about −0.66). This deletion includes *MYO6* gene.

In the present study we identified the second case of UPD ever described associated with HL, in a patient (Sporadic case_4) showing an early-onset bilateral symmetric severe to profound NSHL (Figure 5A). Briefly, TRS revealed a novel homozygous mutation in *LOXHD1* (NM_144612.6) [c.3071A>G; p.(Tyr1024Cys)], apparently segregating only from the father. The variant was predicted as damaging by all *in silico* predictor tools and affected the LH2 domain of the protein. SNP array analysis identified one run of homozygosity bigger than 8 Mb in length, spanning *LOXHD1* gene on chromosome 18. Analysis of informative SNPs in parental samples and their comparison with the patient's genotype confirmed the presence of a paternal UPD. Moreover, a deep analysis of SNPs on the whole chromosome 18 confirmed the presence of both the small isodisomy segment spanning the *LOXHD1* gene plus the presence of heterodisomy on the remaining parts of Chr18 (Figure 5B).

#### OTOA

In Sporadic case_1, showing early-onset bilateral symmetric severe to profound medium-high frequencies hearing loss, a homozygous missense variant [c.1865T>A; p.(Leu622His), rs750007142] in *OTOA* gene (NM_144672.3) was detected. The variant, predicted as damaging by all *in silico* predictor tools, apparently segregated only from the father, thus the clinical case was further investigated by SNP array. Data analysis led to the identification of a large (~228.5 Kb) heterozygous submicroscopic 16p12.2 deletion inherited from the mother (Fontana et al., 2017).

#### *STRC* and *CATSPER2*

In Familial Case_6 and Sporadic Case_15 two different deletion involving *STRC* gene (NM_153700.2) have been identified (Figures 5C–F). The first one belongs to an AR family composed of 4 members, 2 affected siblings (a 5-year old girl and a 3-year old boy) and their normal hearing parents. Both children showed a bilateral moderate symmetric SNHL characterized by a pre-lingual onset (Figure 5C). A 49.23 Kb deletion on chromosome 15 spanning through *STRC* and *CATSPER2* (NM_172095.2) genes was identified (Figure 5D). In the second case, a 10 y.o. girl affected by early-onset bilateral symmetric moderate NSHL, with no familiarity for HL (Figure 5E), an homozygous deletion of 49 Kb involving only *STRC* gene has been detected (Figure 5F).

#### MYO6

In Familial Case_16 SNP arrays led to the identification of a novel heterozygous deletion affecting *MYO6* gene, known for causing autosomal dominant NSHL. The family is composed of two affected siblings (37 and 35 y.o., respectively), the healthy mother (64 y.o.) and the affected father (80 y.o.). All affected individuals display adult onset bilateral moderate symmetric SNHL (Figure 5G) and carry a deletion of 75.8 Kb in *MYO6* gene (Figure 5H).

Overall, the combination of an accurate clinical characterization, TRS and SNP arrays, led to the identification of the molecular cause of hearing loss in 31% of cases (37% of familial cases and 26.3% of sporadic cases). Moreover, among the 14 patients analyzed with SNP array (negative to TRS, or with a single variant in a recessive gene) we reached a molecular diagnosis in ~36% of cases (5 out of 14).

## Discussion

Nowadays, the use of targeted re-sequencing in routine clinical diagnosis seems to be one of the most accurate approaches for the molecular diagnosis of highly heterogeneous genetic diseases, such as inherited deafness. Nevertheless, this approach has some limitations, not being able to accurately detect rearrangements such as deletions and duplications (i.e., CNVs) which might be involved in causing a significant proportion of genetic disorders (Yang et al., 2013). Moreover, recent studies have also suggested that runs of homozygosity (ROH) are much more frequent than previously recognized and, in some cases, can unravel UPD (Wang et al., 2015).

To overcome this limitation and to further increase the detection rate of HHL cases, we refined our strategy performing high-density SNP arrays in TRS-negative cases. Results confirmed that a multi-step integrated approach based on TRS followed by SNP arrays, is extremely powerful in advancing the molecular characterization of HHL. In particular, the largest study of Italian HHL patients so far carried out, demonstrates the importance of SNP arrays analysis in detecting the first case of UPD in *LOXHD1* gene and confirming the importance of genomic rearrangements in the etiopathogenesis of hearing loss.

Combining TRS and SNP arrays, our strategy allowed to characterize 31% of the ~65% of cases negative to *GJB2* mutations (leading to an overall detection rate -including *GJB2*– of ~51%). These results seem to be in agreement with previous data reported by Shearer and Smith (2015) and Sloan-Heggen et al. (2015) in which a detection rate of 39% (including *GJB2* gene) in a much larger cohort is described. Moreover, results of the present study demonstrated a higher detection rate in familial cases (37%), mainly characterized by autosomal dominant inheritance, compared to sporadic cases (26.3%). Also in this case, these data were in agreement with those reported in the literature (71% for familial cases and 37% for sporadic cases) (Shearer et al., 2011; Sloan-Heggen et al., 2016).

Our study highlights the importance of *TECTA* as the second most frequently mutated gene in the Italian population, following *GJB2*. Patients affected by *TECTA* mutations display an early onset symmetrical NSHL, with different degrees of severity (from mild-moderate, to severe-profound). According to literature data, we identified different genotype-phenotype correlations depending on the inheritance pattern and on which of the functional domain of TECTA was affected (Hildebrand et al., 2011). Thus, as expected, Sporadic Case_8, affected by autosomal recessive NSHL, carried a splicing and a non-sense variant, and displayed a moderately severe to severe hearing phenotype (Asgharzade et al., 2017). As regards dominant families, the phenotype of the patients was much more variable and not always reflecting data of previous studies leading to new genotype-phenotype links to be further investigated (Choi et al., 2014).

Another gene frequently mutated in our cohort of patients turned out to be *ACTG1*. Two out of the three mutations identified in this gene were detected as “*de novo*.” So far, several mutations in *ACTG1* gene have been described and analyzed by protein modeling (van Wijk et al., 2003; Rendtorff et al., 2006) although *de novo ACTG1* variants seem to be a rare cause of NSHL (Wang et al., 2018). In this light, we further investigated their potential role by protein modeling and molecular dynamics simulations demonstrating a significant modification of two functional regions of the protein, the tropomyosin, and myosin binding sites and the D-loop. Considering that the binding of tropomyosin and myosin to actin is a key mechanism for regulating the normal function of the complex (Rayment et al., 1993; Behrmann et al., 2012) and that an alteration in actin filament regulation is an important factor in deafness caused by *ACTG1* mutations, these *de novo* variants likely alter the protein activity, leading to deleterious effects (Lee et al., 2018).

Furthermore, another relevant finding of this study is the high prevalence of X-linked forms, which are expected to account for an inconsiderable part of the genetic forms (Smith et al., 2005) and that have been detected in three cases of both dominant and recessive X-linked NSHL, involving *SMPX* and *POU3F4* genes. Among them, a careful attention should be directed to *SMPX* gene whose mutations have been recently reported to lead to a mild bilateral HL phenotype in females and a severe to profound early-onset HL in the affected males (Niu et al., 2017). Alternatively, as in the case of our family, the mother shows a monolateral severe to profound (sky-slope) HL while the proband displays a post-lingual bilateral symmetric severe to profound medium-high frequencies NSHL, as described in only one case worldwide (Weegerink et al., 2011). For this reason, during genetic counseling, it would be very important to carefully verify the monolateral clinical manifestations in affected females as an indicator of an X-linked form of HL.

Finally, our data highlight the huge importance of CNVs discovery in TRS negative cases, or in patients carrying only one mutated allele in recessive genes. In fact, among the 14 families analyzed by SNP arrays, a causative CNV was identified in 5 of them, explaining ~36% of cases.

In particular, this approach allowed the detection of a paternal UPD in chromosome 18, involving *LOXHD1* gene. This represents the second example of UPD associated to NSHL, in addition to that one described in *GJB2*, the most common mutated HL gene. In fact, the only cases of UPD so far described as causative of hearing loss, affect *GJB2* gene (Yan et al., 2007).

Furthermore, CNVs analysis confirmed the significant contribution of *STRC* deletions in hearing loss as recently described in other populations (Plevova et al., 2017). *STRC* deletions are reported in approximately 1% of mixed deafness populations (Francey et al., 2012; Hoppman et al., 2013), making it as a major contributor to congenital hearing impairment, and can cause autosomal recessive NSHL (Verpy et al., 2001) or Deafness-infertility syndrome (DIS) in males if the adjacent *CATSPER2* gene is also involved in the deletion (Zhang et al., 2009). Considering that in Familial Case_6 the deletion also involved *CATSPER2* gene, it will be important to evaluate future fertility problems in the male sibling (since now he is only 3 years old).

Overall, our results proved that the combination of an accurate clinical evaluation, TRS of known NSHL genes and SNP arrays can effectively enhance, in a cost-effective way, the genetic characterization of NSHL, leading to the identification of new mutations/CNVs and thus helping in the clinical management of patients.

## Author Contributions

AM: samples quality control, data production and analysis, study design assessment; SL: data production and analysis; SC: Copy Number Variation (CNV) analysis; VP: Copy Number Variation (CNV) analysis; MM: data production and analysis; EO: samples recruitment, patients' clinical evaluation; SG: samples recruitment, patients' clinical evaluation; UA: samples recruitment, patients' clinical evaluation; MB: data analysis; PoG: protein modeling and molecular dynamics simulation; MB: data production; FF: patients' clinical evaluation; EG: samples recruitment, patients' clinical evaluation; FS: patients' clinical evaluation; AS: samples recruitment, patients' clinical evaluation; CG: samples recruitment, patients' clinical evaluation; MS: samples recruitment, patients' clinical evaluation; PaG: patients' clinical evaluation, data analysis, study design assessment; GG: data analysis, patients' clinical evaluation, study design assessment. All authors contributed in writing or revising the manuscript.

### Conflict of Interest Statement

The authors declare that the research was conducted in the absence of any commercial or financial relationships that could be construed as a potential conflict of interest.

## Footnotes

^1^[Exome Variant Server, NHLBI GO Exome Sequencing Project (ESP), Seattle, WA]. Available online at: http://evs.gs.washington.edu/EVS/
